# Coordinating phosphorus and jasmonate signaling: PHR1 partners with transcriptional regulators

**DOI:** 10.1093/plcell/koad071

**Published:** 2023-03-10

**Authors:** Ching Chan

**Affiliations:** Assistant Features Editor, The Plant Cell, American Society of Plant Biologists, USA; Department of Life Science, National Taiwan Normal University, Taipei 11677, Taiwan

**Figure 1.**
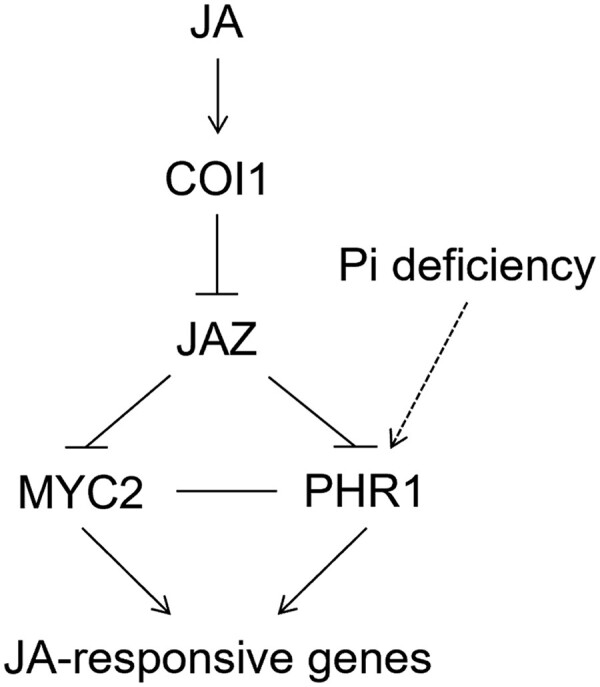
Intersection of PSR and jasmonate signaling. P-deficiency activates PHR1. Jasmonate signaling via COI1 targets JAZ repressors for degradation, enabling MCY2 and PHR1 to accumulate and form a protein complex that positively regulates jasmonate-induced anthocyanin accumulation and root growth inhibition. Reprinted from He et al. (2023), Figure 8.

Phosphorus (P) is one of the essential macronutrients for plant growth and development. PHOSPHATE STARVATION RESPONSE 1 (PHR1) is the central transcriptional regulator that controls a series of plant adaptive strategies to tackle P limitation in natural ecosystems (Paz-Ares et al. 2021). Notably, PHR1 had also been shown to intersect with phytohormone signaling, including jasmonate (Khan et al. 2016) and salicylic acid (Castrillo et al. 2017). These observations expanded the scope of PHR1 function beyond nutrient homeostasis. However, the signaling components that lead to diverged downstream physiological processes remain largely unexplored. **In new work, Kunrong He and colleagues (He et al. 2023)** focused on PHR1-dependent jasmonate signaling and identified a set of partner transcriptional regulators specific for the P-deficiency-induced jasmonate response (see Fig. 1).

PHR1 encodes a member of the MYB transcription factor superfamily, conserved between Arabidopsis (*Arabidopsis thaliana*) and Chlamydomonas (*Chlamydomonas reinhardtii*) (Rubio et al. 2001). There are 4 PHR1 homologs in the Arabidopsis genome, designated PHR1-LIKE1-4 (PHL1-4) (Bustos et al. 2010). In terms of the phosphate starvation response (PSR), PHR1 and PHL1 control much of the transcriptional activation and repression (Bustos et al. 2010), while PHL2 and PHL3 also play redundant roles (Sun et al. 2016; Wang et al. 2023). Interestingly, PHR1, PHL2, and PHL3 were shown to interact with multiple JAZ repressors. Double and triple mutants of these 3 genes (*PHR1*, *PHL2*, *PHL3*) produced a similar phenotype of attenuated P-deficiency-induced anthocyanin accumulation and root growth inhibition, suggesting that PHLs function in various physiological processes in addition to PSR. Furthermore, PHR1 interacted with MYC2/3/4. Mutation of MYCs, as seen in *myc2-1* and *myc234* mutants, also attenuated P-deficiency-induced anthocyanin accumulation and root growth inhibition, similar to that of PHR1, PHL2, and PHL3 mutation. Using genetic crossing, chromatin immunoprecipitation, and transient transcriptional activation assays, the authors delineated the P-deficiency-induced jasmonate signaling pathway and concluded that PHR1 works in concert with the MYCs, which are inhibited by JAZ repressors (see Fig. 1).

Of note, other known regulators of PSR, including PHT1, PHF1, and RNS1, were shown to play insignificant roles in P-deficiency-induced jasmonate signaling (He et al. 2023). This highlights the distinct nature of these signaling pathways, although some signaling components are shared. SPX proteins and inositol pyrophosphates were shown to play pivotal roles in integrating PSR and other metabolic pathways (Lv et al. 2014; Puga et al. 2014; Ried et al. 2021). Their roles in jasmonate signaling might provide further insight into the crosstalk between phosphorus, hormone response, and other plant physiological processes.
